# Case Report: *Hymenolepis diminuta* in an asymptomatic Ecuadorian child.

**DOI:** 10.12688/f1000research.155856.1

**Published:** 2024-10-03

**Authors:** Zulbey Rivero de Rodriguez, Ader Ponce, Anthony Vera, Angela Bracho, Anita Murillo

**Affiliations:** 1 Biological sciences, Universidad Tecnica de Manabi Facultad de Ciencias de la Salud, Portoviejo, Manabí Province, 130105, Ecuador; 2 Laboratory, Vital&Med Clinical Laboratory, Guayaquil, Guayas, Ecuador; 3 Laboratorio Clínico, Universidad Estatal del Sur de Manabi Unidad Academica de Ciencias de la Salud, Jipijapa, Manabí Province, Ecuador

**Keywords:** Hymenolepis diminuta, asymptomatic condition, Child, Preschool, Ecuador

## Abstract

**Background:**

The cestode *Hymenolepis diminuta* is a cosmopolitan parasite, which in the adult stage is usually found in the small intestine of rats and accidentally in humans.

**Case report:**

We describe the finding of eggs of this parasite in an asymptomatic 3-year-old child. The child had extremely high IgE values of 1,376 IU/ml. After receiving treatment with Albendazole suspension 400mg/20mL, on the 10th day post-treatment, he showed no *H. diminuta* eggs in his fecal matter

**Conclusions:**

Detailed morphological review of *Hymenolepis nana*-like eggs is recommended to distinguish them from *H. diminuta* eggs.

## Introduction

The cestode *Hymenolepis diminuta* (Rudolphi 1819, Weinland 1858) is a cosmopolitan parasite, which in the adult stage is usually found in the small intestine of rats and accidentally in humans. Its biological cycle develops with the intervention of an arthropod, which becomes infected by ingesting the eggs of *H. diminuta*, which are found in the feces of parasitized rats. ^1^

The mechanism of infection begins when coprophilic arthropods ingest the eggs of *H. diminuta* eliminated by rodent feces and act as obligate intermediate hosts of the cestode. When humans accidentally ingest these infected arthropods, the cysticercoids present in the insect’s hemocele develop to adults in the human’s small intestine, where eggs are then produced and expelled in defecation. Human infection through direct ingestion of *H. diminuta* eggs has not been reported to date. ^2^

Several coprophilous arthropods such as fleas, weevils, lepidoptera and beetles have been described as intermediate hosts, ^3^ as well as different species of beetles of the genera *Tribolium* and *Tenebrio.* ^4^ Experimentally, it has been shown that 90 species of arthropods can serve as intermediate hosts. ^5^ Although, Joyeux ^6^ pointed to *Tenebrio molitor, Ceratophyllus fasciatus* and *Xenopsylla cheopis* as the usual sources of rat infestation, the beetles of the genus *Tribolium* are those indicated by the scientific literature, as the most frequent in rat and human infections.

The adult cestode of *H. diminuta* is 20 to 60 cm long, its scolex is rounded and small with four cup-shaped suckers, it has a rostellum without hooks, which is invaginated in a cavity located in the most apical portion of the scolex. The strobilar chain contains three characteristic portions of proglottids: immature, mature, and gravid; The latter, when detached from the strobilus, disintegrate and release the eggs that are eliminated along with the feces. ^7^ The eggs are rounded, 60 to 80 microns in size, yellowish in color with a thick outer membrane and a smaller oncosphere inside, with three pairs of hooks and no polar filaments. ^8^

Clinically, as in *Hymenolepis nana* infection, parasitosis can be asymptomatic. In infections with a high parasite load, the most frequent symptoms of *H. diminuta* hymenolepiasis are digestive, with abdominal pain and diarrhea prevailing; irritability and anal itching may also occur ^2, 3, 9^ and in some cases patients with skin conditions have presented. ^10, 11^

It is an uncommon infection in humans; however, the prevalence is high in some places: 7.8% in schoolchildren between 6 and 15 years of age in Magdalena in Cajamarca ^12^ and 7.1% in the population of Tingo María in Huánuco in Peru. ^13^ It can be deduced that food, stored and in contact with rats and beetles, when consumed constitutes the means of infection, by ingestion of infected beetles. There are few cases in the literature that report infections in adults ^3^ since most have been described in children, ^2, 9– 11, 14– 17^ probably due to ingesting the infected arthropods without realizing it. However, a 2020 review of *H. diminuta* ^18^ states that, out of 130 infections in humans, the age of the infected individuals was: 2.3% infants (≤1 year old), 66.2% children (2 to 9 years), 20% adolescents (10 to 19 years old), and 11.5% were adults (≥ 20 years old).

Regarding geographical location, the review by Panti-May et al. ^18^ reports that human infections with *H. diminuta* were reported in 80 countries around the world from 1810 to 2018. Most cases were reported in the Americas (n = 815), Southeast Asia (n = 226) and Eastern Mediterranean (n = 210). Three countries exhibited more than 100 records: Costa Rica (n = 320), the United States of America (n = 181), and Brazil (n = 140).

In Ecuador, the presence of *Hymenolepis* spp. has been described ^19^ in stray dogs on the coastal beaches of Ecuador, *Hymenolepis nana* and *diminuta* have also been reported in rodents ( *Rattus norvegicus* and *Rattus rattus*) from the city of Milagro. ^20^ Only one case has previously been reported of this species in a two-year-old Ecuadorian girl, however, the finding was made in Spain ^11^; This case describes that in 2003 it arrived in that continent and in the first half of 2004 it presented gastrointestinal symptoms, detecting the parasite in feces. In 2007, research by Jacobsen et al. ^21^ indicated a 1% prevalence of *H. diminuta* when evaluating 203 samples from children between 12 and 60 months of age; however, no reference was made to clinical or sociodemographic data of the infected child (ren). Since then, no other case of infection in humans has been described in the country, so the case of an asymptomatic child from Portoviejo, Ecuador, is presented.

## Case report

**Figure 1.**
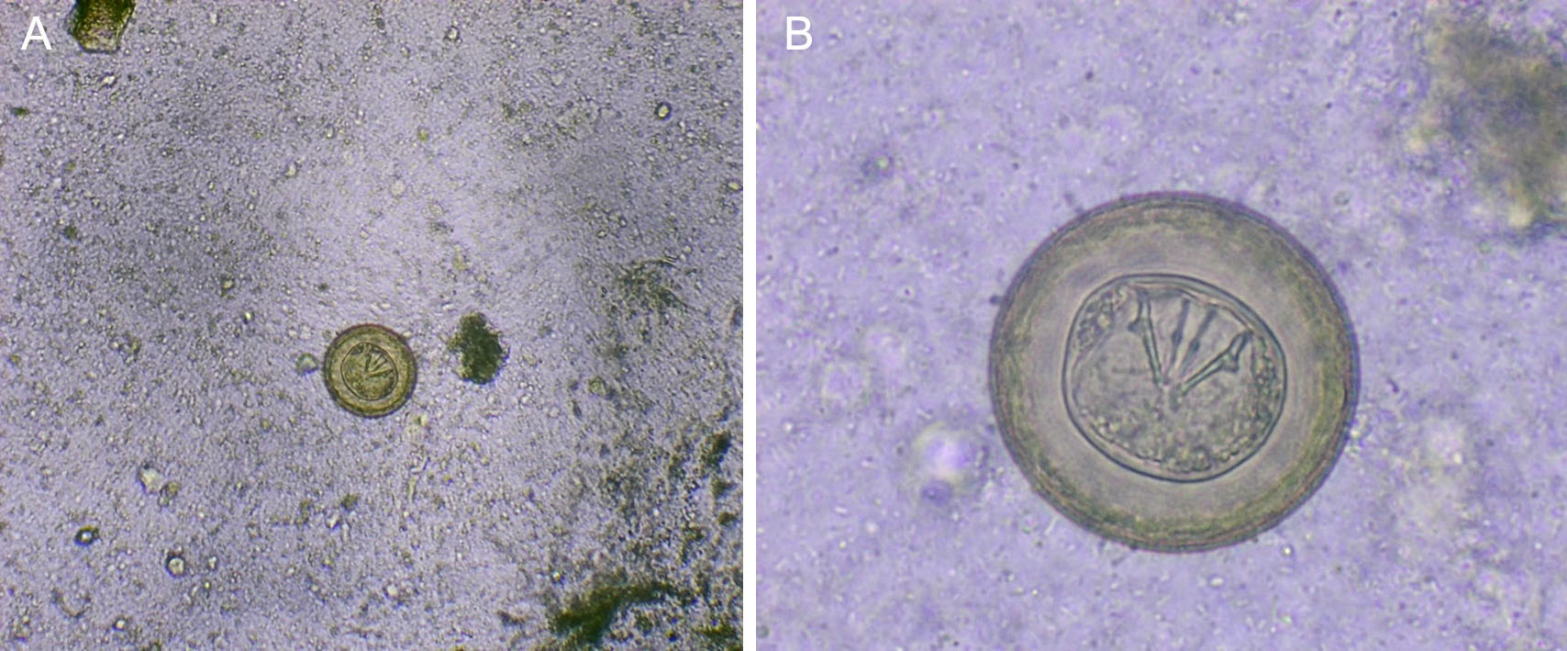
(A) *H. diminuta* eggs at 100 × magnification, physiological saline; (B) Eggs of *H. diminuta* at 400 × magnification, physiological saline.

In February 2024, the stool sample of a 3-year-old child is received at the Parasitology laboratory of the Technical University of Manabí. On macroscopic examination, the sample showed a soft, greenish-brown consistency, without mucus, pus, or blood; There are also no obvious food residues. Microscopic examination with physiological saline, showed eggs morphologically compatible with eggs of *Hymenolepis diminuta* ( Figure 1A,B). These had their characteristic brown color, large size and absence of polar filaments. They were measured with a micrometer scale, where 4 of them measured 60 microns and 1 measured 70 microns in diameter.

When the father is consulted, he indicates that the child did not have gastrointestinal symptoms and that the stool test was carried out as part of the annual health evaluation that he performs. When reviewing the rest of the tests performed, it was observed in the hematology: Leukocyte count within the reference values: 7.15×102/μl (5.0 to 10.00), but with a decrease in neutrophils: 1.59×102/μl (2.10 to 8.90), an increase in lymphocytes: 4.64×102/μl (1.30 to 4.60) and a slight increase in eosinophils: 5.2% (0.0 to 4.0). Hemoglobin 12.5 g/dl (10.5 to 13.5 g/dl), Hematocrit: 36.0% (32.0 to 40.0%). The results show that, although C-reactive protein was within the reference values: 0.33 mg/L (0.00 to 5.00 mg/L), there was a significant increase in the value of Immunoglobulin E (IgE): 1.376 IU/ml (0.0 to 60.0 IU/ml).

To assess the child’s living conditions, his home was visited. The house is in a peri-urban community of the city of Portoviejo (Colón parish). Although Colón is considered an urban parish, the house is in a wooded area of the parish, and in the back has an abundance of intricate trees. In general terms, the house is neat and has the basic services of water, electricity, and septic tank, for the disposal of excreta. There is a chance of rats and mice in the back wooded area, which is ideal for synanthropic animals. During the visit, the presence of beetles (genus *Hexodon*) is observed and since there are dogs, the presence of fleas, which have been described as intermediate hosts of *H. diminuta*, cannot be ruled out.

The child received anthelmintic treatment with Albendazole suspension 400 mg/20 mL, 2 bottle, one per day. On the 4th day after treatment, a post-treatment stool examination was performed and eggs were still observed, which were quite altered in their morphology and difficult to identify. Subsequently, 10 days after the end of treatment, the stool examination showed no evolutionary forms of parasites.

## Discussion

*Hymenolepis diminuta* infection is a cosmopolitan zoonosis that is mainly rural and infrequent in humans. As of 2020, 1,561 published records of *H. diminuta* infection had been identified in 80 countries ^18^; mainly reported in children. ^2, 9– 11, 14– 17^

Although the child in this case did not present clinical manifestations and most of his laboratory tests were within the reference ranges, the high IgE value found is remarkable. The child does not suffer from allergies, so this situation was ruled out as the cause of the increase. Helminth infections have been mainly related to blood eosinophilia ^22– 24^ and a similar situation has only been reported in terms of increased IgE in a child with *H. diminuta* in Italy ^10^ who presented atypical allergic manifestations. ^11^ In this research, it is pointed out that the infected 2-year-old child presented remitting fever (maximum peak 37.7 °C), abdominal pain, diffuse skin itching, transient chest rash and arthromyalgia and in the same context, the case of the Ecuadorian girl in Spain is presented, who manifested atopic dermatitis, self-limited diarrhea, vomiting and constipation, unlike the current case. there were no allergic manifestations or any symptoms, only the elevation of IgE.

Allergic symptoms are often present in chronic helminth infections, caused by a constant state of immune activation characterized by a dominant type of Th2 cytokine profiles and high IgE levels. ^25, 26^ However, eosinophilia is not usually seen in helminth infections that reside in the lumen of the human intestine, as is the case with *H. diminuta.* However, helminth infections can affect the expression of an allergic disease and, in certain situations, may be associated with a higher, lower, or no risk of atopic conditions. ^27, 28^

Another important point to highlight is the anthelmintic treatment applied, since the literature reports that the first choice anthelmintics are praziquantel and niclosamide, ^3^ therefore, it has been the treatment of choice in several studies. ^9– 11, 14– 17^ However, in this case, albendazole was indicated, a treatment also applied in a case reported by Rivero et al. ^2^ in Venezuela, demonstrating effectiveness in the treatment of parasitosis, as no eggs were observed in successive samples. It is known that first-choice drugs are given in single doses, however, Tena et al. ^14^ indicated in their study to repeat the dose three times, even increased, until the parasite was eliminated, so albendazole can be considered as an alternative treatment.

## Conclusions

It is feasible to find *H. diminuta* infection in Ecuadorian children, although in an infrequent way, so the microscopist should be alert to the finding of eggs like those of *Hymenolepis nana*, but larger and dark brown in fresh preparations. Likewise, medical personnel should not rule out the possibility of this parasitic species in children, mainly under five years of age.

### Ethics and consent

Written informed consent for publication of their clinical details and/or clinical images was obtained from the parent relative of the patient. Written informed consent has been obtained from the father of the patient to publish this paper. Informed assent could not be obtained, because the child was very young (3 years old).

## Data Availability

No data are associated with this article.
