# Supplementary material for: Case Report: Hymenolepis diminuta in an asymptomatic Ecuadorian child
Source: F1000Res. 2025 Feb 20;13:1119. Originally published 2024 Oct 3. [Version 3] doi: 10.12688/f1000research.155856.3 (PMC11822246; doi:10.12688/f1000research.155856.3)
Supplement: Peer review history

# Reviewer report: Reviewer response for version 2

**DOI:** 10.5256/f1000research.174667.r361809

**Published:** 2025-02-12

**Authors:** Amal Sm Sayed

**Affiliations:** 1 Head of Zoonoses Department, Assiut University, Asyut, Assiut Governorate, Egypt

The article is highlights the importance of *H.diminuta *infection in human and health implications. They provided background on *H.diminuta*. infection and their epidemiology are sufficient. The case study was well-written and the figures support the results mentioned by the authors. However, some corrections still should be made to improve the article. The correction are provided in the PDF file: *https://f1000research.s3.amazonaws.com/linked/708089.h_rev..pdf*

Are enough details provided of any physical examination and diagnostic tests, treatment given and outcomes?

Yes

Is the case presented with sufficient detail to be useful for other practitioners?

Yes

Is sufficient discussion included of the importance of the findings and their relevance to future understanding of disease processes, diagnosis or treatment?

Yes

Is the background of the case’s history and progression described in sufficient detail?

Yes

Reviewer Expertise:

Zoonoses, Microbiology, Infectious diseases

I confirm that I have read this submission and believe that I have an appropriate level of expertise to confirm that it is of an acceptable scientific standard, however I have significant reservations, as outlined above.

# Response

**Published:** 2025-02-12

**Authors:** Zulbey Rivero de Rodriguez

**Affiliations:** Biological Sciences, Universidad Tecnica de Manabi, Portoviejo, Manabí Province, Ecuador

Greetings, I will proceed to make the text corrections that were suggested.

# Reviewer report: Reviewer response for version 2

**DOI:** 10.5256/f1000research.174667.r341287

**Published:** 2024-11-22

**Authors:** Adriana Esteves

**Affiliations:** 1 University of the Republic Uruguay, Montevideo, Montevideo Department, Uruguay

I have no further comments.

Are enough details provided of any physical examination and diagnostic tests, treatment given and outcomes?

Yes

Is the case presented with sufficient detail to be useful for other practitioners?

Yes

Is sufficient discussion included of the importance of the findings and their relevance to future understanding of disease processes, diagnosis or treatment?

Yes

Is the background of the case’s history and progression described in sufficient detail?

Yes

Reviewer Expertise:

Molecular parasitology

I confirm that I have read this submission and believe that I have an appropriate level of expertise to confirm that it is of an acceptable scientific standard.

# Reviewer report: Reviewer response for version 1

**DOI:** 10.5256/f1000research.171080.r331924

**Published:** 2024-11-04

**Authors:** Adriana Esteves

**Affiliations:** 1 University of the Republic Uruguay, Montevideo, Montevideo Department, Uruguay

       The Case Report: *Hymenolepis diminuta* in an asymptomatic Ecuadorian child from Rivero and coworkers is very interesting in that it shows an atypical case of a parasitosis in a patient.

 The data provided in the introduction is correct and very appropriate.

 Recommendations to the authors:

  

- Explain the meaning of “direct ingestion of eggs” I understand that its different from accidentally, but it is difficult to understand a voluntary ingestion.
- “… probably due to ingesting the infected arthropods without realizing it.” The explanation is not convincing.
- The indicated period of reports around the world is too long to have an idea of ​​the evolution of the disease. It would be appropriate to indicate, for example, the current prevalence in each country mentioned, or at least some way that allows such old cases to be evidenced by recent ones.
- What’s the meaning of (ren) at the end of a sentence from the last paragraph of the introduction.
- IgE data after treatment would be an important input complementing the microscopic evaluation. If you have them, I suggest including them
- “… unlike the current case. there were no allergic manifestations ….“Typing error, there should be a comma instead of a period.

Are enough details provided of any physical examination and diagnostic tests, treatment given and outcomes?

Yes

Is the case presented with sufficient detail to be useful for other practitioners?

Yes

Is sufficient discussion included of the importance of the findings and their relevance to future understanding of disease processes, diagnosis or treatment?

Yes

Is the background of the case’s history and progression described in sufficient detail?

Yes

Reviewer Expertise:

Molecular parasitology

I confirm that I have read this submission and believe that I have an appropriate level of expertise to confirm that it is of an acceptable scientific standard.

# Response

**Published:** 2024-11-10

**Authors:** Zulbey Rivero de Rodriguez

**Affiliations:** Biological Sciences, Universidad Tecnica de Manabi, Portoviejo, Manabí Province, Ecuador

1.-When direct ingestion of eggs is indicated, it means ingestion of the eggs as such, not in any other evolutionary way. It means that even if an egg is swallowed, infection has not been described. It is necessary to ingest the cysticercoid found inside an arthropod.If you think it is strictly necessary to explain this better, please let me know.

 2.-In the part where it is indicated that children are more susceptible to infection, it was changed without realizing it, because without paying attention to it.

 3.- The placement of child(ren) was to indicate the plural of child, but it was changed at once to children

 4.-We do not have IgE data from the child after treatment, as far as we know only stool tests were done again (we did them)

 5.-Corrected, the period (dot) was removed and a comma was placed

# Reviewer report: Reviewer response for version 1

**DOI:** 10.5256/f1000research.171080.r331922

**Published:** 2024-10-28

**Authors:** Yukifumi Nawa

**Affiliations:** 1 Khon Kaen University, Khon Kaen, Thailand

General:

 Although this manuscript is a case report of asymptomatic *Hymenoleepis diminuta* infection in a child in Ecuador, actual content of this manuscript is a kind of the mixture of a case report and a insufficient review of *H. diminuta* infection in general. Thus, as a case report, the entire manuscript is lengthy and wordy with too much of unnecessary reference citation.

 Specific:

 Introduction

 This is a report of an asymptomatic hymenolepiasis case. Thus, this case has little significance/attractive issues in terms of clinical practice. Also, as is mentioned by the authors, *H. diminuta* infection in humans, especially among children of poor sanitary condition has been sporadically but repeatedly reported from various parts of the world.

 The only merit/appeal of this case report is the confirmed case of *H. diminuta *infection in a child in Ecuador, where the presence of such a parasitic disease is ignored by the clinical practitioners in the country. Thus, the first 4 paragraphs of general description of *H. diminuta *infection should be merged into one short introductory paragraph.

 Case report

 The 3 years old boy has received regular health checking and his stool sample was examined in the Parasitology Laboratory. However, his stool was examined only by direct microscopic observation for the saline-diluted specimen (Please give more data as to how much of stool with what degree of saline-dilution). What about other coproparasitological examinations? What about possible co-infection with other intestinal helminths or protozoa?

 Discussion

 The same problem as those seen in the Introduction. The Discussion part of the case report should be limited to the findings and the problems and the tasks directly related to the present case. As it stands, the Discussion section of this manuscript contains too much of general information not directly related to this cs.

 Conclusions

 As it stands, the Conclusion is too general. Conclusions of the case report should be specific from the observations of this case.

Are enough details provided of any physical examination and diagnostic tests, treatment given and outcomes?

Partly

Is the case presented with sufficient detail to be useful for other practitioners?

Yes

Is sufficient discussion included of the importance of the findings and their relevance to future understanding of disease processes, diagnosis or treatment?

Partly

Is the background of the case’s history and progression described in sufficient detail?

Partly

Reviewer Expertise:

Parasitology

I confirm that I have read this submission and believe that I have an appropriate level of expertise to confirm that it is of an acceptable scientific standard, however I have significant reservations, as outlined above.

# Response

**Published:** 2024-11-10

**Authors:** Zulbey Rivero de Rodriguez

**Affiliations:** Biological Sciences, Universidad Tecnica de Manabi, Portoviejo, Manabí Province, Ecuador

1.-The reviewer's suggestion is respected, but changes were made to the introduction, as it is considered short enough that it is necessary to eliminate information that is important to provide to the reader.

 2.- Information on stool tests is included, because if we had the concentrate result and forgot to put that in the article

 3.- The discussion further discussed the increase in eosinophils and IgE in cases of H. diminuta, such as this one. It was added that unfortunately there was no data on post-treatment IgE.

 4.- Something else is added for conclusion, taking into account the reviewer's suggestion
